# Associations between the Levels of Estradiol-, Progesterone-, and Testosterone-Sensitive MiRNAs and Main Clinicopathologic Features of Breast Cancer

**DOI:** 10.3390/jpm12010004

**Published:** 2021-12-21

**Authors:** Tatiana Kalinina, Vladislav Kononchuk, Efim Alekseenok, Grigory Abdullin, Sergey Sidorov, Vladimir Ovchinnikov, Lyudmila Gulyaeva

**Affiliations:** 1 Federal Research Center of Fundamental and Translational Medicine, Timakova Str. 2/12, 630117 Novosibirsk, Russia; kononchuk@niimbb.ru (V.K.); alekseenok@niimbb.ru (E.A.); gulyaeva@niimbb.ru (L.G.); 2 Meshalkin National Medical Research Center, Ministry of Health of the Russian Federation, Rechkunovskaya Str. 15, 630055 Novosibirsk, Russia; 3 Novosibirsk Regional Oncological Dispensary, Plakhotnogo Str., 2, 630000 Novosibirsk, Russia; grabdullin@mail.ru; 4 Department of Breast Pathology, Novosibirsk Municipal Publicly Funded Healthcare Institution Municipal Clinical Hospital #1, Zalessky Str. 6, 630047 Novosibirsk, Russia; svsidorov@yandex.ru; 5 Institute of Cytology and Genetics of Siberian Branch of the Russian Academy of Sciences, Prospekt Lavrentyeva 10, 630090 Novosibirsk, Russia; tdkaliki@gmail.com; 6 Institute for Medicine and Psychology, Novosibirsk State University, Pirogova Str. 2, 630090 Novosibirsk, Russia

**Keywords:** microRNA, breast cancer, biomarker, lymph node metastasis, hormone-dependent carcinogenesis

## Abstract

Despite the existing advances in the diagnosis and treatment of breast cancer (BC), the search for markers associated with the clinicopathological features of BC is still in demand. MiRNAs (miRs) have potential as markers, since a change in the miRNA expression profile accompanies the initiation and progression of malignant diseases. The receptors for estrogen, androgen, and progesterone (ER, AR, and PR) play an important role in breast carcinogenesis. Therefore, to search for miRNAs that may function as markers in BC, using bioinformatic analysis and the literature data, we selected 13 miRNAs whose promoter regions contain binding sites for ER or AR, or putative binding sites for ER, AR, and PR. We quantified their expression in MCF-7 cells treated with estradiol, progesterone, or testosterone. The levels of miRNAs sensitive to one or more of these hormones were quantified in BC samples (*n* = 196). We discovered that high expression levels of miR-190b in breast tumor tissue indicate a positive ER status, and miR-423 and miR-200b levels differ between patients with and without HER2 amplification. The miR-193b, -423, -190a, -324, and -200b levels were associated with tumor size or lymph node status in BC patients, but the presence of these associations depended on the status and expression level of ER, PR, HER2, and Ki-67. We also found that miR-21 expression depends on HER2 expression in ER- and/or PR-positive BC. The levels of miRNA were significantly different between HER2 0 and HER2 1+ tumors (*p* = 0.027), and between HER2 0 and HER2 2+, 3+ tumors (*p* = 0.005).

## 1. Introduction

The choice of treatment for breast cancer (BC) depends on the expression level of the estrogen and progesterone receptors (ER and PR), HER2 (a receptor for epidermal growth factor), and Ki-67. The levels of these proteins in tumor tissues are determined by immunohistochemistry (IHC). However, IHC has several disadvantages, such as type and duration of tissue fixation, the choice of antibody, and the experience of the pathologist, all of which affect the reproducibility of the results [1]. Axillary lymph node (ALN) status is another important factor in the diagnosis of BC that predicts disease-free survival and overall survival. Conventional methods for diagnosing lymph node metastases (LNM) at the preoperative stage, such as ultrasound, mammography, and magnetic resonance imaging (MRI), have relatively low accuracy and sensitivity [2]. In recent years, sentinel lymph node biopsy (SLNB) has been used to diagnose lymph node status. However, not all medical facilities have the ability to conduct SLNB. In addition, the false-negative rate of the procedure is estimated between 4.6% and 16.7%, which also restricts the popularity of this method [3]. Therefore, in many regions of Russia, axillary lymph node dissection (ALND) is performed to diagnose metastatic lesions of the lymph nodes. Both SLNB and especially ALND can lead to a number of complications. Thus, despite the existing advances in the diagnosis and treatment of BC, the search for new markers associated with the clinicopathological features of BC is still relevant. MiRNAs (miRs) have great potential as markers, since a change in the miRNA expression profile accompanies the initiation and progression of malignant diseases.

ER, PR, and androgen receptor (AR) play important roles in breast carcinogenesis, targeting the regulators of cell cycle, signaling, differentiation, and apoptosis [4,5,6]. MiRNAs can also be targets of these receptors [7,8]. Therefore, to search for miRNA-markers of breast carcinogenesis using bioinformatics analysis and the literature data, we selected some miRNAs potentially regulated by ER, AR, or PR. We examined their levels in MCF-7 cells treated with estradiol, progesterone, and testosterone. The levels of those miRNAs that significantly responded to hormone treatment were analyzed in BC samples (*n* = 196).

## 2. Materials and Methods

### 2.1. Cell Culture

MCF-7 cells were obtained from the Russian Cell Culture Collection (St. Petersburg (Russia) branch of the ETCS). Cells were cultivated in Iscove’s modified Dulbecco’s medium (IMDM; Gibco BRL Co., Gaithersburg, MD, USA) with 10% of FBS (Gibco BRL Co., Gaithersburg, MD, USA), 2 mM L-alanyl-L-glutamine (Gibco BRL Co., Gaithersburg, MD, USA), 250 mg/mL amphotericin B, and 100 U/mL penicillin/streptomycin (Gibco BRL Co., Gaithersburg, MD, USA). The cells were maintained in a 5% CO2 incubator at 37 °C. The medium was refreshed every 2–3 days, and the cells were passaged when 65–80% confluent. The absence of mycoplasma contamination was verified by conventional PCR assays. At 48 h prior to the addition of hormones, the culture medium was changed to phenol red-free IMDM (Gibco BRL Co., Gaithersburg, MD, USA) to eliminate the weak estrogen-agonistic activity of phenol red. Estradiol, progesterone, and testosterone (Sigma-Aldrich, St. Louis, MO, USA) were dissolved in dimethyl sulfoxide (DMSO; Sigma-Aldrich, USA), and then the solutions were diluted with the culture medium so that the final DMSO concentration was 0.1% (*v*/*v*). Cells treated with 0.1% DMSO were used as a control. The cells were treated for 6, 24, or 48 h.

### 2.2. Tissue Samples

A total of 196 pairs of BC tissue samples and samples of normal adjacent tissue from female patients who had not received preoperative pharmacotherapy, were collected between 2017 and 2020 at Novosibirsk municipal publicly-funded healthcare institution Municipal Clinical Hospital #1 and Novosibirsk Regional Oncological Dispensary. Tissue samples were placed in an RNAlater™ Stabilization Solution (Invitrogen™, Carlsbad, CA, USA) and kept at −20 °C until experiments were performed. Clinicopathologic information was obtained by reviewing medical records and reports on results of immunohistochemical assays. The following variables were determined: the T stage, N stage; IHC scores on ER, PR, HER2, and Ki-67 (Table 1). For cases with HER2 IHC-score 2+, the determination of the final HER2 status was carried out using FISH. All patients recruited into the study had grade 2 (G2) tumors. Breast cancer subtypes were categorized according to the St. Gallen Expert Consensus as follows [9]: luminal A (ER^+^ and/or PR^+^, HER2^−^, and Ki-67 < 14%), luminal B HER2-negative (ER^+^ and/or PR^+^, HER2^−^, and Ki-67 ≥ 14%) luminal B HER2-positive (ER^+^ and/or PR^+^, HER2^+^), HER2-positive (ER^−^, PR^−^, and HER2^+^), and triple-negative (ER^−^, PR^−^, and HER2^−^).

### 2.3. MicroRNA Isolation

Total miRNA was extracted from human tissue by a previously published protocol [11].

### 2.4. MiRNA Reverse Transcription and Real-Time PCR (RT-PCR)

Relative expression levels for miRNAs were measured by real-time reverse transcription-PCR. A reverse-transcription reaction was carried out using stem-loop primers [12] and the RT-M-MuLV-RH kit (Biolabmix Ltd., Novosibirsk, Russia). Real-time PCR was performed with TaqMan probes and the BioMaster UDG HS-qPCR (2×) kit (Biolabmix Ltd.). To detect PCR products, a CFX96™ Detection System (Bio-Rad Laboratories, Hercules, CA, USA) was applied. Small nuclear RNAs U44 and U48 were used to normalize the data.

Primers for the reverse transcription were as follows: hsa-miR-190a-5p, 5′-GTCGTATCCAGTGCAGGGTCCGAGGTATTCGCACTGGATACGACACCTAATA-3′; hsa-miR-190b-5p, 5′-GTCGTATCCAGTGCAGGGTCCGAGGTATTCGCACTGGATACGACAACCCAA-3′; hsa-miR-23a-3p, 5′- GTCGTATCCAGTGCAGGGTCCGAGGTATTCGCACTGGATACGACGGAAATC -3′; hsa-miR-27a-3p, 5′-GTCGTATCCAGTGCAGGGTCCGAGGTATTCGCACTGGATACGACTGCTCACA -3′; hsa-miR-193b-3p, 5′-GTCGTATCCAGTGCAGGGTCCGAGGTATTCGCACTGGATACGACAGCGGGAC-3′; hsa-miR-324-5p, 5′-GTCGTATCCAGTGCAGGGTCCGAGGTATTCGCACTGGATACGACACACCAAT-3′; hsa-miR-423-3p, 5′-GTCGTATCCAGTGCAGGGTCCGAGGTATTCGCACTGGATACGACACTGAGGG-3′; hsa-miR-200b-3p, 5′-GTCGTATCCAGTGCAGGGTCCGAGGTATTCGCACTGGATACGACGTCATCAT-3′; hsa-miR-21-5p, 5′-GTCGTATCCAGTGCAGGGTCCGAGGTATTCGCACTGGATACGACTCAACATC-3′; hsa-miR-126-3p, 5′- GTCGTATCCAGTGCAGGGTCCGAGGTATTCGCACTGGATACGACCGCATTAT -3′; hsa-miR-378a-3p, 5′- GTCGTATCCAGTGCAGGGTCCGAGGTATTCGCACTGGATACGACGCCTTCT -3′; hsa-miR-149-5p, 5′- GTCGTATCCAGTGCAGGGTCCGAGGTATTCGCACTGGATACGACGGGAGTGA -3′; hsa-miR-342-3p, 5′- GTCGTATCCAGTGCAGGGTCCGAGGTATTCGCACTGGATACGACACGGGTG -3′; U44, 5′-GTCGTATCCAGTGCAGGGTCCGAGGTATTCGCACTGGATACGACAGTCAGTT-3′; U48, 5′-GTCGTATCCAGTGCAGGGTCCGAGGTATTCGCACTGGATACGAGACGGTCAG-3′.

The following specific oligonucleotides were employed for RT-PCR: hsa-miR-190a-5p, (forward primer) 5′-GCCGCTGATATGTTTGATA-3′, (probe) 5′-(R6G)-TTCGCACTGGATACGACACCTAATA-(BHQ1)-3′; hsa-miR-190b-5p, (forward primer) 5′-GCCGCTGATATGTTTGATA-3′, (probe) 5′-(R6G)-TTCGCACTGGATACGACAACCCAA-(BHQ1)-3′; hsa-miR-23a-3p, (forward primer) 5′-GCCGCATCACATTGCCAGG-3′, (probe) 5′-(R6G)-TTCGCACTGGATACGACGGAAATC-(BHQ1)-3′; hsa-miR-27a-3p, (forward primer) 5′-GCCGCTTCACAGTGGCTAA-3′, (probe) 5′-(R6G)-TTCGCACTGGATACGACGCGGAAC-(BHQ1)-3′; hsa-miR-193b-3p, (forward primer) 5′-GCCGCAACTGGCCCTCAAA-3′, (probe) 5′-(R6G)-TTCGCACTGGATACGACAGCGGGAC-(BHQ1)-3′; hsa-miR-324-5p, (forward primer) 5′-CCCGCATCCCCTAGGGC-3′, (probe) 5′-(R6G)-TTCGCACTGGATACGACACACCAAT-(BHQ1)-3′; hsa-miR-423-3p, (forward primer) 5′-GCCGAGCTCGGTCTGAGGC-3′, (probe) 5′-(R6G)-TTCGCACTGGATACGACACTGAGG-(BHQ1)-3′; hsa-miR-200b-3p, (forward primer) 5′-GCCGCTAATACTGCCTGGTA-3′, (probe) 5′-(R6G)-TTCGCACTGGATACGACGTCATCAT-(BHQ1)-3′; hsa-miR-21-5p, (forward primer) 5′-GCCGCTAGCTTATCAGACT-3′, (probe) 5′-(R6G)-TTCGCACTGGATACGACTCAACATC-(BHQ1)-3′; hsa-miR-126-3p, (forward primer) 5′-GCCGCTCGTACCGTGAGTA-3′, (probe) 5′-(R6G)-TTCGCACTGGATACGACCGCATTAT-(BHQ1)-3′; hsa-miR-378a-3p, (forward primer) 5′-GCCGCACTGGACTTGGAGTC-3′, (probe) 5′-(R6G)-TTCGCACTGGATACGACGCCTTCT-(BHQ1)-3′; hsa-miR-149-5p, (forward primer) 5′-GCCGTCTGGCTCCGTGTCT-3′, (probe) 5′-(R6G)-TTCGCACTGGATACGACGGGAGTGA-(BHQ1)-3′; hsa-miR-342-3p, (forward primer) 5′-GCCGCTCTCACACAGAAATCG-3′, (probe) 5′-(R6G)-TTCGCACTGGATACGACACGGGTGC-(BHQ1)-3′; U44, (forward primer) 5′-GCCGCTCTTAATTAGCTCT-3′, (probe) 5′-(R6G)-TTCGCACTGGATACGACAGTCAGTT-(BHQ1)-3′; U48, (forward primer) 5′-CCCTGAGTGTGTCGCTGATG-3′, (probe) 5′-(R6G)-TTCGCACTGGATACGAGACGGTCAG-(BHQ1)-3′. A similar type of reverse primer targeting the stem-loop region in the synthesized cDNAs was 5′-AGTGCAGGGTCCGAGGTA-3′. Each sample was analyzed in triplicate. Relative expression level was assessed based on threshold cycle (Ct) values taking into account PCR efficacy (E) for both the analyzed and reference RNAs.

### 2.5. Bioinformatics Analysis

The list of miRNAs containing ER binding sites in their promoter regions was previously published [13]. To search for miRNAs potentially regulated by AR or PR receptors, putative miRNA promoter regions were extracted from the human genome (hg38) 10,000 nucleotides upstream from the start of a precursor miRNA sequence according to MirGeneDB [14]. AR and PR binding sites were searched in these regions using position weight matrices (MA0007.2, MA0113.3) from Jaspar (http://jaspar.genereg.net/, accessed on 12 December 2017) [15] (sequences of binding sites for these receptors are the same) using Biostrings (R Bioconductor package) [16]. We additionally performed a search for binding sites in the promoter regions of rat miRNAs (using MA0007.1 and MA0113.1). The putative promoter regions were extracted from rat genome (Rnor_6.0), 10,000 nucleotides upstream from the start of a precursor miRNA sequence according to miRBase v21 [17]. For further research, miRNAs were selected that have high expression in breast tissues according to the Human miRNA tissue atlas (https://ccb-web.cs.uni-saarland.de/tissueatlas/, accessed on 17 October 2021) [18].

### 2.6. Statistical Analysis

STATISTICA software (version 12; TIBCO Software Inc., Palo Alto, CA, USA) was used for statistical data analysis and plotting. Data are presented as median values. The Shapiro–Wilk test was used to check data normality. Since the distribution was not normal in some groups, the statistical analysis was carried out using the non-parametric Mann–Whitney U test. Data with *p* < 0.05 were regarded as statistically significant.

## 3. Results

### 3.1. Selection of Estradiol-, Progesterone-, Testosterone-Sensitive miRNAs

The list of microRNAs potentially regulated by ER was published earlier [13]. MiRNAs, whose promoter regions contain sequences corresponding to the AR and PR binding sites, were searched using Biostrings. For the study, we selected miRNAs with high expression in breast tissues. We were interested in miRNAs containing ER binding sites in promoter shared by the three species (human, rat, and mouse), and AR/PR binding sites in promoter in humans and rats (Table 2). Interest in such miRNAs is due, firstly, to the possibility of further studies of the regulation of their expression in vivo, and secondly, because the regulation of miRNA by ER, PR, and AR in different species indicates an essential role of such miRNAs in the signaling pathways of receptors. Thus, we chose miR-21, miR-190b, miR-200b, miR-23a, miR-27a, miR-342, miR-190a, miR-378a, miR-324, miR-423, and miR-149 for analysis. We also took miR-193b and miR-126 into study, since their targets are ER and PR.

The relative levels of selected miRNAs were determined in MCF-7 cells treated with estradiol (E2), progesterone (P4), or testosterone by RT-PCR (Table 3). Treatment of cells with E2 led to a significant change in the expression of miR-190b, miR-200b, and miR-193b. The miR-190b level decreased in cells treated with 100 nM E2 for 6 h, but increased 1.4-fold under the influence of both doses of the hormone after 24 h of incubation. Expression of miR-200b decreased in cells treated with 100 nM E2 for 6 h, and expression of miR-193b increased 1.3-fold after 48 h of incubation of cells with 100 nM E2. For miR-200b, the decrease in its level in MCF-7 cells after 6 h of treatment with E2 was also reported earlier (Table 2).

In cells treated with testosterone, the expression of miR-27a, miR-190a, miR-200b, miR-21, miR-423, miR-193b, and miR-324 significantly changed. After 6 h of incubation with 100 nM testosterone, the levels of miR-27a and miR-21 decreased (1.3- and 1.6-fold, respectively). However, after 48 h, the level of miR-21 increased 1.4-fold under the influence of both doses of testosterone. In addition, in cells treated with 10 or 100 nM testosterone, the levels of miR-190a, miR-200b, miR-423, and miR-193b increased. The level of miR-324 increased only in cells treated with a high dose of testosterone.

Treatment of MCF-7 with P4 led to changes in the levels of miR-190b, miR-190a, miR-21, and miR-324. The level of miR-190b increased 1.3-fold in cells treated with 100 nM P4 for 48 h. The expression of miR-190a decreased by 1.3-times under the action of both doses of P4 after 24 h of incubation. The level of miR-21 increased 1.4-fold after 48 h of incubation of cells with 100 nM P4. Finally, miR-324 expression was significantly increased 1.6- and 2.2-fold in cells treated with low and high doses of P4, respectively, after 6 h of incubation.

Thus, we identified hormone-sensitive miRNAs: miR-190b, miR-193b, miR-324, miR-190a, miR-200b, miR-21, miR-423, and miR-27a.

### 3.2. Analysis of the Hormone-Sensitive MiRNAs Expression in Breast Cancer

**Figure 1:**
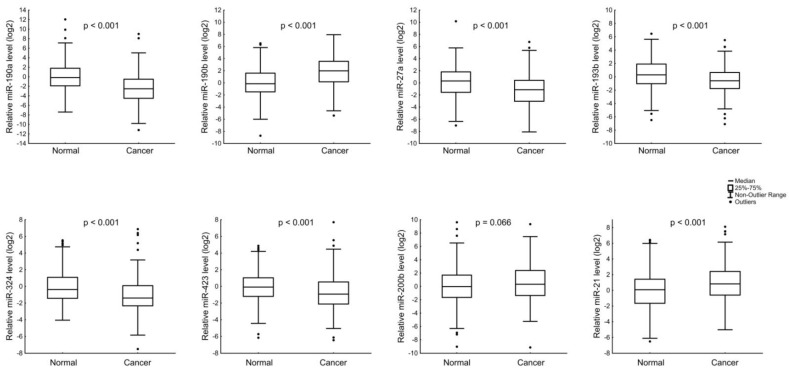
The comparison of miRNA expression between normal and cancerous tissue groups. The Y axis presents the expression level ($\log_{2}2^{–\Delta \Delta \mathrm{Ct}}$).

The relative levels of identified miRNAs were determined in 196 pairs of tumors and healthy tissues by RT-PCR. The amount of miR-190a, miR-27a, miR-193b, miR-324, and miR-423 was reduced in BC tissues compared to normal tissues (Figure 1). In contrast, miR-190b and miR-21 levels were increased in BC.

We investigated whether the expression of an identified miRNA depends on the status of ER, PR, HER2, Ki-67 index, or age. We observed that the amount of miR-190b and miR-21 in tissues depended on the ER and PR status, and the expression of miR-423 and miR-200b depended on the HER2 status (Table 4). The relative level of miR-190b was significantly higher in ER^+^ and/or PR^+^ tumors, and the level of miR-21 was significantly higher in ER^−^ and PR^−^ tumors. The expression levels of miR-423 and miR-200b were significantly higher in the tumors of patients with HER2-amplified cancer than in tumors with HER2 0 and HER2 1+ expression scores (according to IHC). Furthermore, the amount of miR-423 and miR-200b was associated with Ki-67 index. The levels of these miRNAs were higher in tumors with high Ki-67 (≥14%).

Expression of miR-27a and miR-21 was lower in the tumors of patients older than 50 years compared to tumors of younger patients. MiR-193b was found to be associated with the status of the lymph nodes—the amount of miRNA was lower in the tumor tissues of patients with LNM.

### 3.3. Expression of MiR-190a, MiR-190b, MiR-27a, MiR-193b, MiR-324, MiR-423, MiR-200b, and MiR-21 in Relation to Clinicopathologic Features of ER- and/or PR-Positive BC

Next, we evaluated the relation between the expression of miRNAs and the clinicopathologic features of tumors with positive ER and PR status (Table 5). We also analyzed whether there is a relation between miRNA counts and tumor characteristics within specific BC subtypes. In the analysis, we separately considered the group of patients with the HER2 1+ expression score. HER2 expression is higher in HER2 1+ BC compared to HER2 0 tumors [34]. As previously shown, HER2 1+ and HER2 0 tumors differ in the expression profile of a number of genes, and clinically, HER2-low (i.e., 1+ and lack of *ERBB2* amplification) BC shows more ALN involvement compared to HER2 0 BC [35]. We have also previously demonstrated that the relation of miRNA levels with tumor characteristics can be different for these variants of tumors [36].

**Figure 2:**
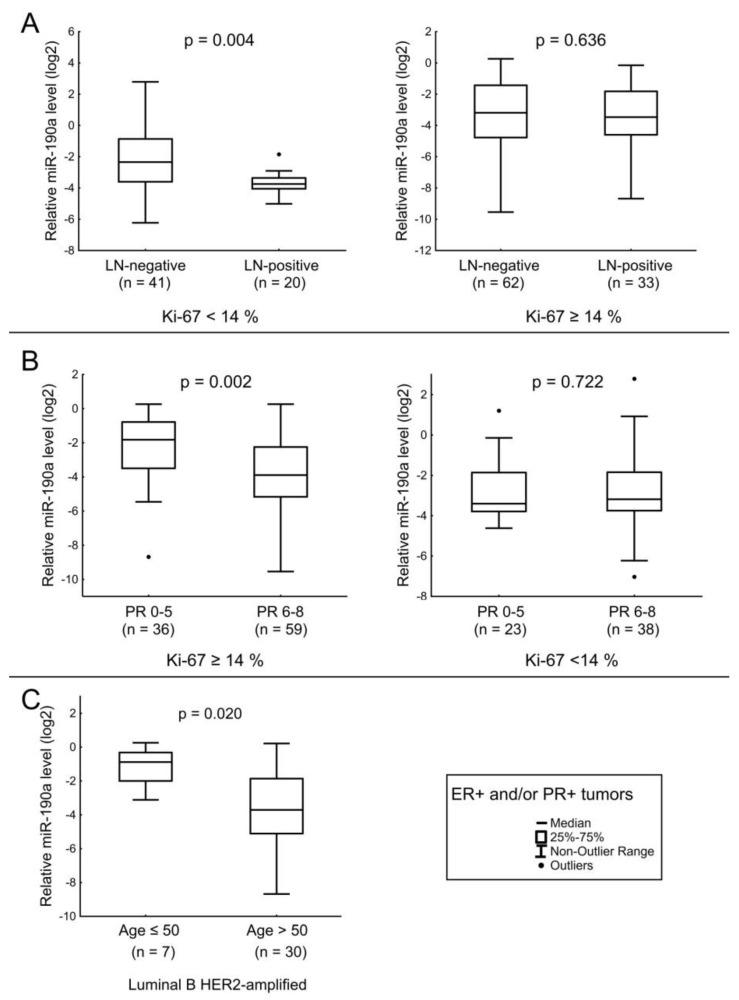
The comparison of miR-190a expression between different cancerous tissue groups: (**A**) relation of miR-190a level with the LNM in tumors with Ki-67 < 14% (left), relation of miR-190a level with the LNM in tumors with Ki-67 ≥ 14% (right); (**B**) relation of miR-190a level with the PR expression level in tumors with Ki-67 ≥ 14% (left), relation of miR-190a level with the PR expression level in tumors with Ki-67 < 14% (right); (**C**) relation of miR-190a level with age of patients with luminal B HER2-aplified BC. The Y axis presents the expression level ($\log_{2}2^{–\Delta \Delta \mathrm{Ct}}$); the results were normalized to the control (normal tissue).

For miRNA-190a, a nearly significant tendency towards a decrease in its level was observed in the tissues of patients with LNM compared to cases without LNM. Detailed analysis revealed that for tumors with Ki-67 < 14%, the decrease in the level of miR-190a in the presence of metastases was significant (Figure 2A). The miR-190a relative level was decreased in tumor tissues of patients with high PR levels compared to those with PR IHC scores of 0–5. However, this association with the PR level was not observed in tumors with a Ki-67 < 14% (Figure 2B). Additionally, the amount of miRNA was decreased in the BC tissues of patients over 50 years old compared to younger patients with the luminal B HER2-amplified BC subtype (Figure 2C).

**Figure 3:**
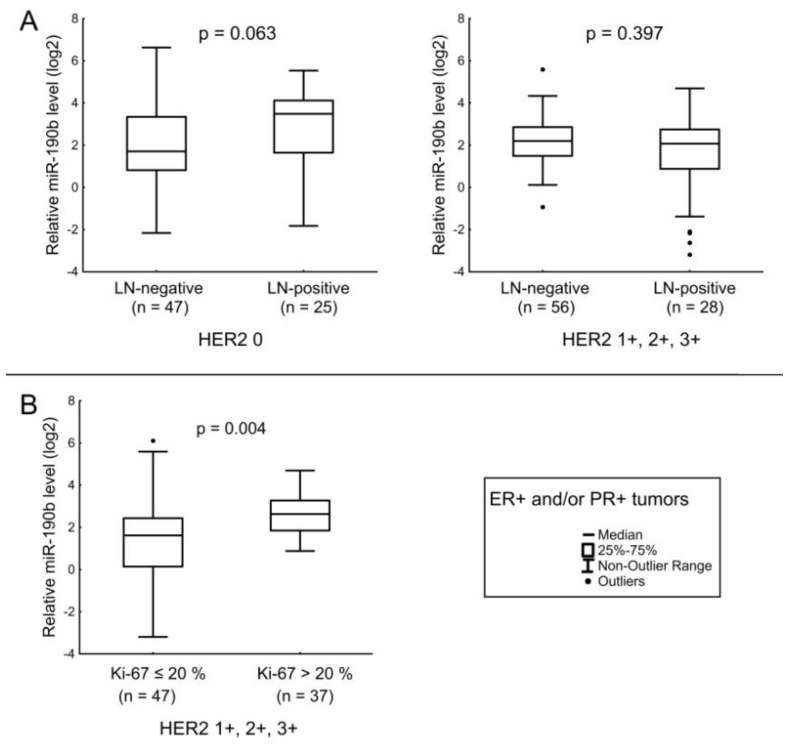
The comparison of miR-190b expression between different cancerous tissue groups: (**A**) relation of miR-190b level with the LNM in tumors with HER2 IHC score 0 (left), relation of miR-190b level with the LNM in tumors with HER2 IHC score 1+, 2+, 3+ (right); (**B**) relation of miR-190b level with Ki-67 in tissues of patients with luminal HER2-expressing BC (20% was the median value of Ki-67 index). The Y axis presents the expression level ($\log_{2}2^{–\Delta \Delta \mathrm{Ct}}$); the results were normalized to the control (normal tissue).

For miR-190b, there was a nearly significant tendency to an increase in its level in tumor tissues of patients with LNM in HER2-non-expressing BC (HER2 0) (Figure 3A). In HER2-expressing BC (HER2 score 1+, 2+, 3+), the miRNA level was increased in the tissues of patients with Ki-67 levels above the median compared to cases with lower Ki-67 (Figure 3B).

**Figure 4:**
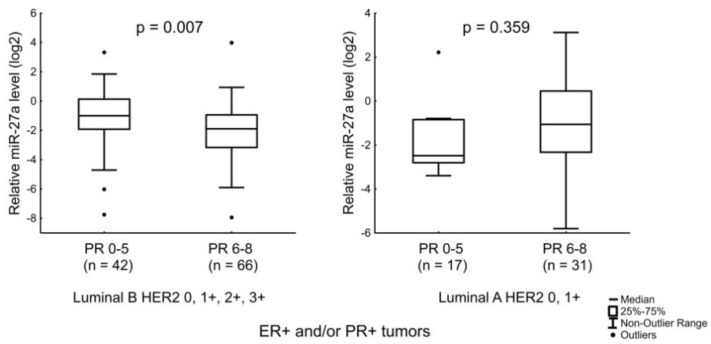
Relation of miR-27a level with PR expression level in luminal B tumors (left) and relation of miR-27a level with PR expression level in luminal A tumors (right). The Y axis presents the expression level ($\log_{2}2^{–\Delta \Delta \mathrm{Ct}}$); the results were normalized to the control (normal tissue).

For miR-27a, we noted the presence of an association with the level of PR expression. Detailed analysis showed that in the luminal B subtypes, the miRNA level was reduced in tumor tissues of patients with a high PR level (6–8 IHC score) compared to tissues of patients with a lower level of PR (Figure 4).

**Figure 5:**
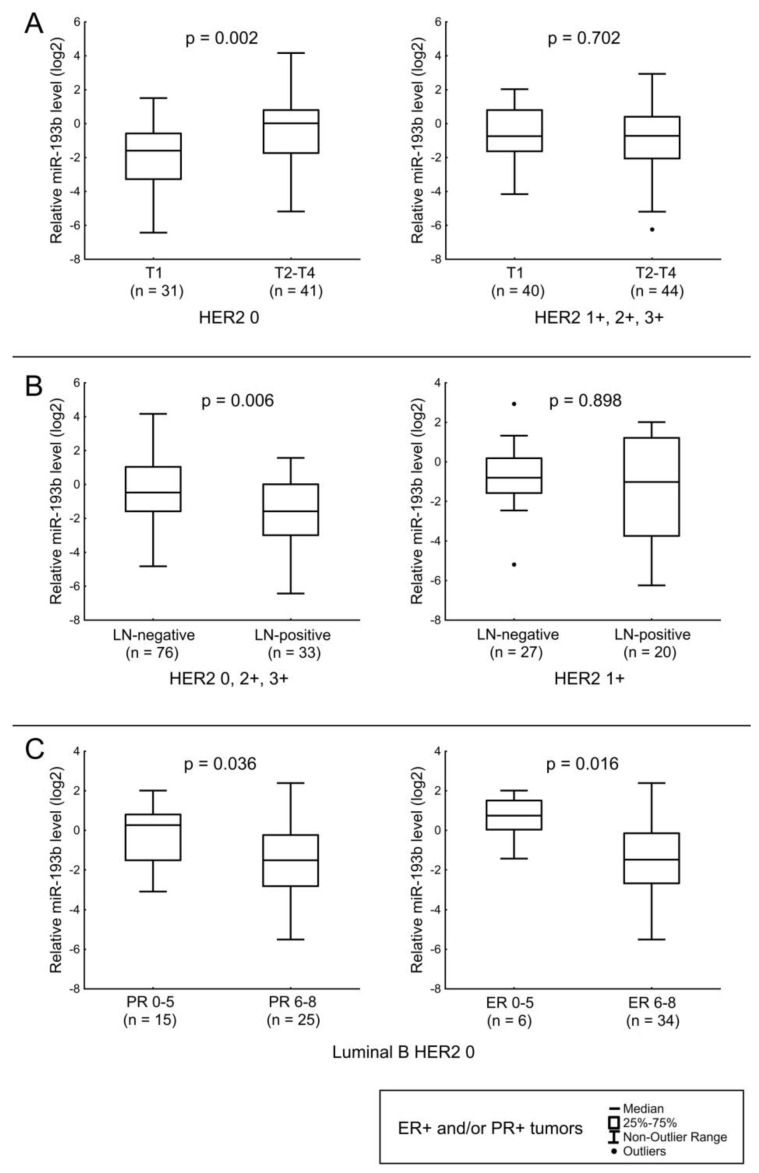
The comparison of miR-193b expression between different cancerous tissue groups: (**A**) relation of miR-193b level with the T stage in tumors with HER2 IHC score 0 (left), relation of miR-193b level with the T stage in tumors with HER2 IHC score 1+, 2+, 3+ (right); (**B**) relation of miR-193b level with LNM in tumors with HER2 IHC score 0, 2+, 3+ (left), relation of miR-193b level with LNM in tumors with HER2 IHC score 1+ (right); (**C**) relation of miR-193b level with PR and ER expression levels in luminal B HER2 0 BC. The Y axis presents the expression level ($\log_{2}2^{–\Delta \Delta \mathrm{Ct}}$); the results were normalized to the control (normal tissue).

For miR-193b, an association was found with the presence of LNM, the level of ER expression, and we also observed a tendency for its level to increase with increasing tumor size. The relation with tumor size was found to be significant for HER2-non-expressing tumors (Figure 5A). A lower level of miR-193b in tumor tissues of patients with LNM was observed in all cases, except for tumors with HER2 1+ expression (Figure 5B). Since most cases with low ER expression belong to the luminal B HER2 0 variant, we separately assessed the association of miRNA with ER and PR expression in this BC type (Figure 5C).

**Figure 6:**
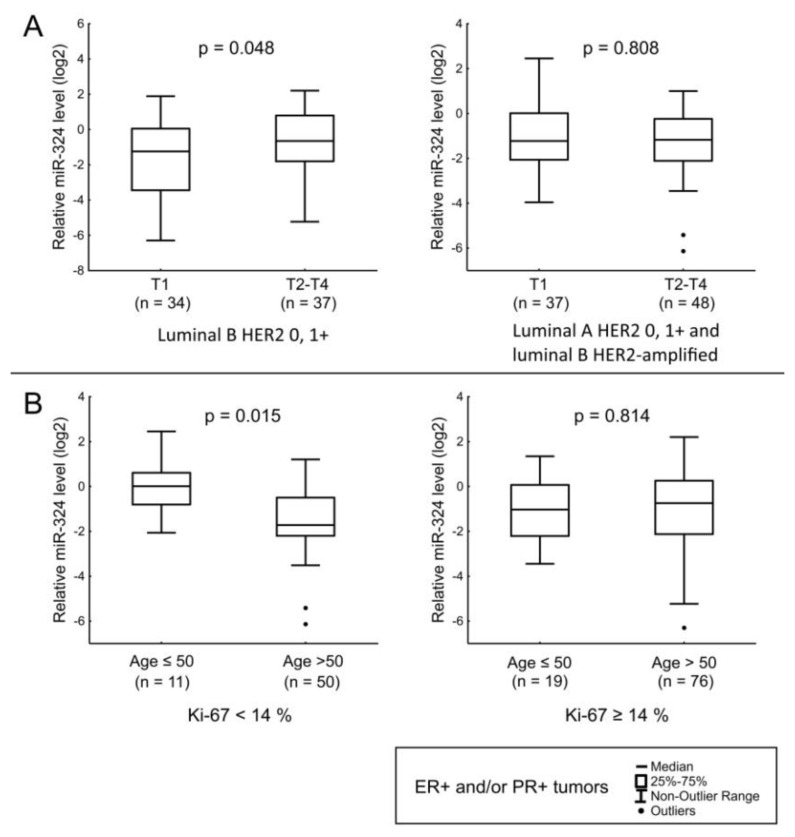
The comparison of miR-324 expression between different cancerous tissue groups: (**A**) relation of miR-324 level with the T stage in luminal B HER2-non-amplified tumors (left), relation of miR-324 level with the T stage in luminal A and luminal B HER2-amplified tumors (right); (**B**) relation of miR-324 level with age of patients in tumors with Ki-67 < 14% (left), relation of miR-324 level with age of patients in tumors with Ki-67 ≥ 14% (right). The Y axis presents the expression level ($\log_{2}2^{–\Delta \Delta \mathrm{Ct}}$); the results were normalized to the control (normal tissue).

MiR-324 levels have been found to be associated with tumor size in luminal B HER2-non-amplified BC (Figure 6A). Furthermore, the amount of miRNA was lower in the tumors of patients over 50 years old compared to younger patients in cases of the disease with Ki-67 < 14% (Figure 6B).

**Figure 7:**
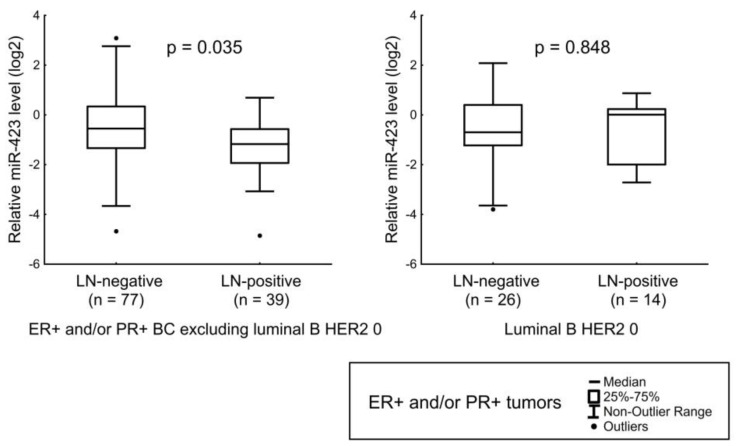
Relation of miR-423 level with LNM in luminal A, luminal B HER2-amplified, and luminal B HER2 1+ tumors (left); relation of miR-423 level with LNM in luminal B HER2-non-expressing tumors (right). The Y axis presents the expression level ($\log_{2}2^{–\Delta \Delta \mathrm{Ct}}$); the results were normalized to the control (normal tissue).

We observed that the level of miR-423 is reduced in tumor tissues of patients with LNM. This decrease was not observed only for patients with luminal B HER2 0 BC (Figure 7).

**Figure 8:**
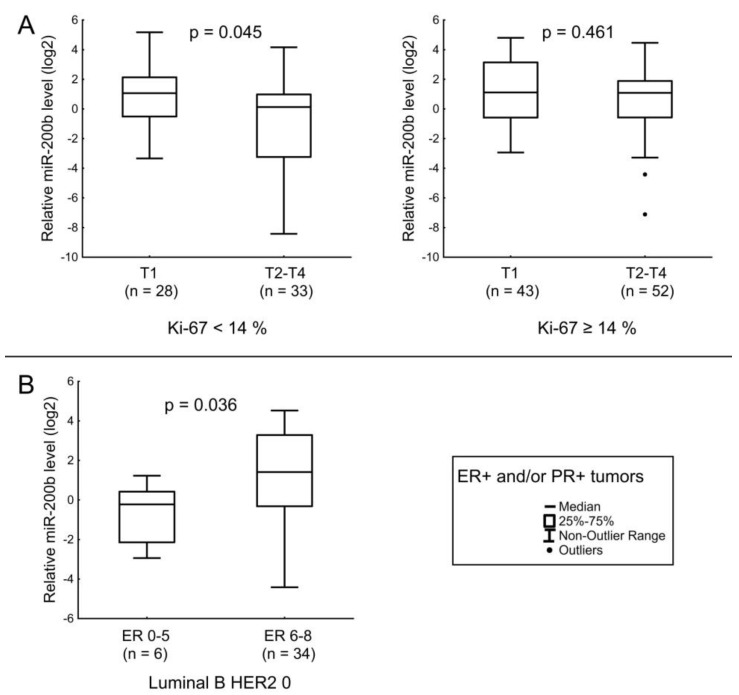
The comparison of miR-200b expression between different cancerous tissue groups: (**A**) relation of miR-200b level with the T stage in tumors with Ki-67 < 14% (left), relation of miR-200b level with the T stage in tumors with Ki-67 ≥ 14% (right); (**B**) relation of miR-200b level with ER expression level in luminal B HER2-non-expressing tumors. The Y axis presents the expression level ($\log_{2}2^{–\Delta \Delta \mathrm{Ct}}$); the results were normalized to the control (normal tissue).

In patients with tumors with Ki-67 < 14%, the miR-200b level was lower in cases with tumors > 2 cm (Figure 8A). Additionally, when analyzing the general sample of patients with ER^+^ and/or PR^+^ it was found that the level of miR-200b is associated with the level of ER. In Figure 8B, we showed the association of the miRNA level with ER expression only for patients with luminal B HER2 0 BC.

**Figure 9:**
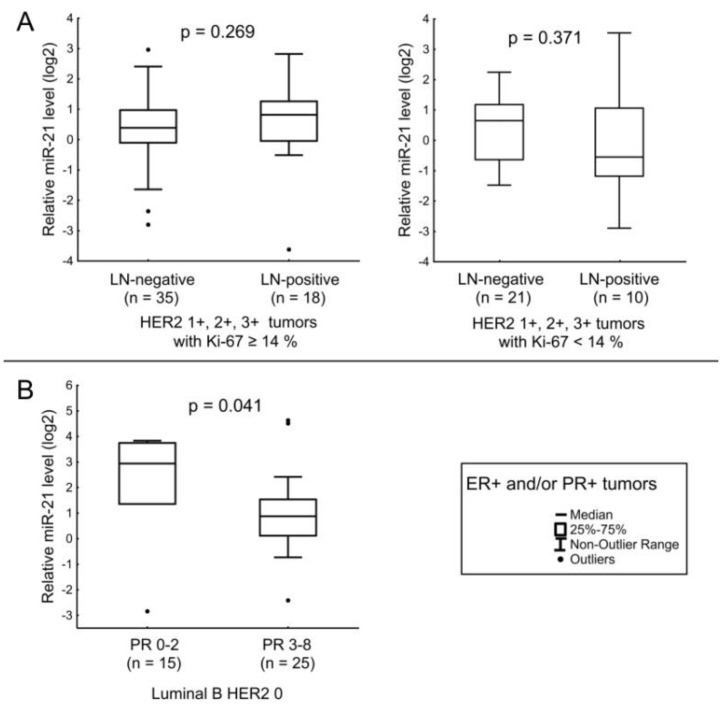
The comparison of miR-21 expression between different cancerous tissue groups: (**A**) relation of miR-21 level with LNM in HER2-expressing tumors with Ki-67 ≥ 14% (left), relation of miR-21 level with LNM in HER2-expressing tumors with Ki-67 < 14% (right); (**B**) relation of miR-21 level with PR expression level in luminal B HER2-non-expressing tumors. The Y axis presents the expression level ($\log_{2}2^{–\Delta \Delta \mathrm{Ct}}$); the results were normalized to the control (normal tissue).

For miR-21, there was a tendency to an increase in its level in cases with LNM for patients with ER^+^ and/or PR^+^ HER2-expressing tumors with Ki-67 ≥ 14% (Figure 9A). In luminal B HER2-non-expressing tumors, the amount of miR-21 was significantly lower in the BC tissues of patients with PR IHC scores of 3–8 than in patients with IHC scores of 0–2 (Figure 9B).

We also analyzed whether there is a relation between the HER2 level in the tumor, determined using IHC, and the expression levels of the studied miRNAs. We found that in tumors with Ki-67 < 14%, miR-324 levels were significantly higher in cases where HER2 amplification was confirmed, compared to cases without HER2 amplification (*p* = 0.019).

**Figure 10:**
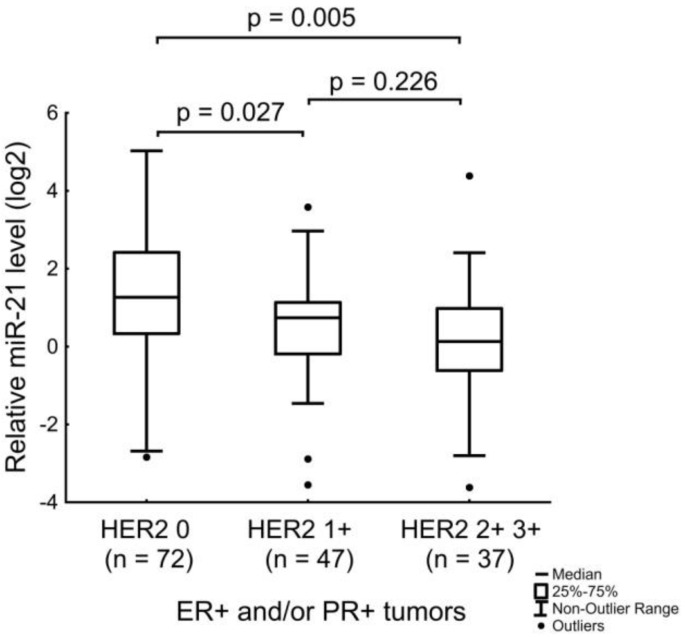
The relation of the miR-21 level with the HER2 expression level in ER-positive and/or PR-positive tumors. The Y axis presents the expression level ($\log_{2}2^{–\Delta \Delta \mathrm{Ct}}$); the results were normalized to the control (normal tissue).

The amount of miR-21 in tumor tissue was significantly different in patients with a HER2 level estimated at a 0 score according to the IHC, from the amount of miRNA in patients with HER2-amplified BC or HER2 1+ BC (Figure 10).

**Figure 11:**
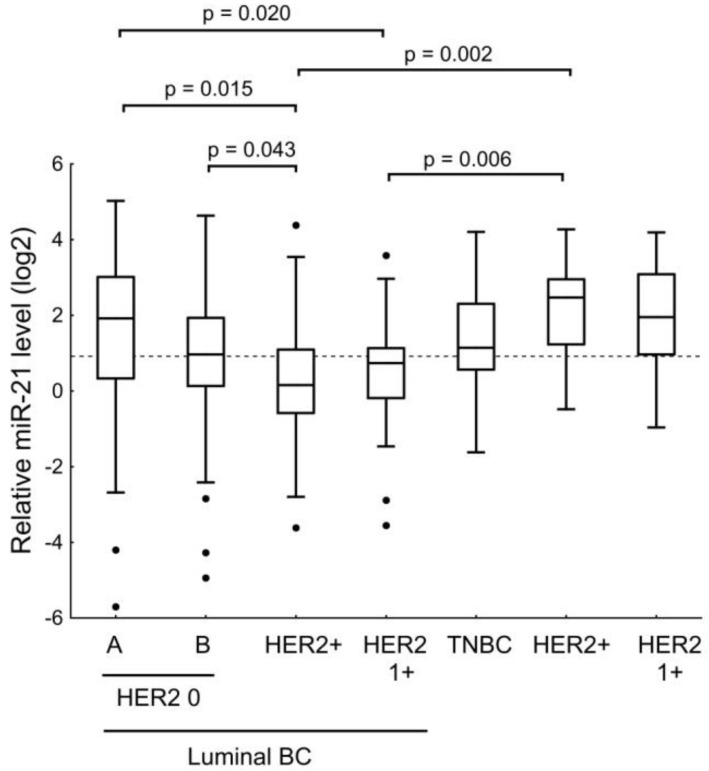
The comparison of miR-21 expression between different BC subtypes. HER2+ = HER2 amplification. Tumors with HER2 IHC scores of 1+ were treated as a separate group. The Y axis presents the expression level ($\log_{2}2^{–\Delta \Delta \mathrm{Ct}}$); the results were normalized to the control (normal tissue).

Since an association with the level of HER2 expression was found for miR-21, we analyzed its level in the BC subtypes (Figure 11). We found that miR-21 level in luminal A HER2 0 tumors was significantly different from the level of this miRNA in luminal B HER2^+^ BC and luminal tumors with HER2 1+ expression levels. The expression of miRNA-21 in the tissues of the luminal B HER2 0 BC was significantly different only in comparison with its level in the tissues of the luminal HER2-amplified BC. However, we found that in ER- and PR-negative tumors, the highest level of miR-21 was in HER2-expressing tumors. In addition, the levels of miRNA were significantly differentiated between luminal B HER2-amplified cancer and ER- and PR-negative HER2-amplified BC, and between luminal HER2 1+ cancer and HER2-amplified BC.

### 3.4. Expression of MiR-190a, MiR-190b, MiR-27a, MiR-193b, MiR-324, MiR-423, MiR-200b, and MiR-21 in Relation to Clinicopathologic Features of ER- and PR-Negative BC

Next, we analyzed the relation between the levels of the studied miRNAs and the characteristics of tumors in patients with ER- and PR-negative BC (Table 6). We found that miR-200b levels are significantly increased in BC tissues from patients with tumors > 2 cm. The amount of miR-21 in tumor tissue depended on the age of the patients and was significantly increased in patients under the age of 50.

**Figure 12:**
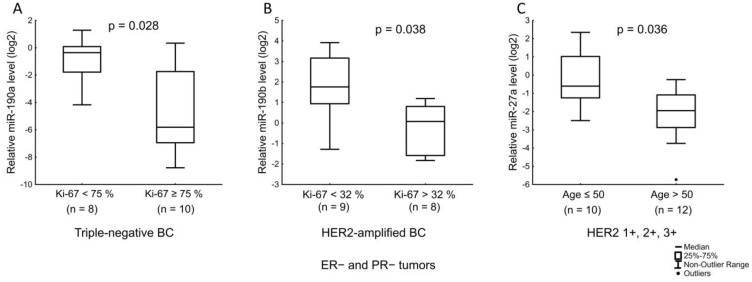
The comparison of miR-190a, miR-190b, and miR-27a expression between different cancerous tissue groups: (**A**) relation of miR-190a level with Ki-67 index in TNBC (75% was the median value of Ki-67); (**B**) relation of miR-190b level with Ki-67 in HER2-amplified tumors (32% was the median value of Ki-67); (**C**) relation of miR-27a level with age of patients with HER2-expressing tumors. The Y axis presents the expression level ($\log_{2}2^{–\Delta \Delta \mathrm{Ct}}$); the results were normalized to the control (normal tissue).

We found that miR-190a was associated with a Ki-67 index in patients with triple-negative BC (TNBC): miR-190a was reduced in tumors with Ki-67 ≥ 75% (Figure 12A). In HER2-amplified BC, miR-190b was reduced in tumors with high Ki-67 (> 32%) (Figure 12B). Finally, miR-27a was found to be associated with the age of patients with HER2-expressing BC (Figure 12C).

## 4. Discussion

The most common cancer in women is BC. In the past decade, significant progress has been made in the diagnosis and treatment of this disease. However, there are still some problems in diagnosing the disease. For example, it was previously noted that there may be a discrepancy between the results of the HER2 expression assessment by IHC and the results of fluorescence in situ hybridization, and the level of this discrepancy is higher in small laboratories [1]. In a recent study, it was shown that the concordance rates between the results of the IHC analysis of samples obtained during biopsy and the results of the IHC analysis of samples obtained during surgery for HER2 and Ki-67 are 84.8% and 83.5%, respectively [37]. In general, this is a good agreement, but it indicates that there may be an insufficient treatment or, conversely, an over-treatment of patients at the preoperative stage. Another problem is that the accuracy and sensitivity of the currently used imaging tests for determining the status of ALN in the preoperative phase are relatively low [2]. Thus, the search for markers that can subsequently serve to clarify the diagnosis or identify metastases is still in demand.

It is known that steroid receptors ER, PR, and AR play an important role in the initiation and progression of BC. Therefore, here, in order to search for markers associated with the clinicopathological features of tumors, we identified miRNAs sensitive to estradiol, progesterone, or testosterone, and analyzed their level in 196 pairs of tumors and healthy breast tissues.

For the study, we selected 13 miRNAs that are highly expressed in breast cells, and whose promoter regions contain sequences corresponding to the ER, PR, and AR binding sites. Of the selected miRNAs, the expression of eight were significantly altered in ER-, PR-, and AR-positive MCF-7 cells under the influence of one or more compounds (Table 7). We further analyzed the levels of identified hormone-sensitive miRNAs in BC samples.

In the analysis, we divided patients into groups depending on ER and PR status, HER2 status, or Ki-67 index. We separately analyzed a group of patients with a HER2 1+ IHC score; as previously shown, biologically, HER2 1+ tumors are significantly different from HER2 0 tumors [34]. In our study, there were no patients with HER2 2+ tumors with a lack of HER2 amplification. The main identified associations between miRNA expression levels and tumor characteristics are presented in Table 8.

We found that the expression of miR-190b and miR-21 depends on the status of ER and PR. The differences between miR-190b levels in tumor tissues with ER IHC scores of 6–8, and in tumors with ER IHC scores of 0–5, were also close to significant. Previously, it was demonstrated that the level of miR-190b is significantly increased in ER-positive breast tumors [22]. Thus, the result of our study is consistent with the previously obtained data. However, the same study reported no change in miR-190b expression in MCF-7 treated with estradiol (incubation with 1 nM E2 for 6, 18, and 96 h). Here, we detected an increase in the miR-190b level in MCF-7 cells treated with 10 nM and 100 nM E2 for 24 h. Thus, given the presence of an ER binding site in the promoter of this miRNA, according to the ChIP-seq data, it can be expected that a high level of miR-190b in ER^+^ and/or PR^+^ breast tumors is due to the increased expression and activity of ER.

As for miR-21, earlier it was shown in smaller samples that its level is higher in ER-positive BC [38,39]. According to our data, the expression of miRNA is higher in tumors with a negative status of ER and PR. However, the observed dependence may be related to HER2. So, we observed the lowest level of miR-21 expression in ER^+^ and/or PR^+^ HER2-amplified and ER^+^ and/or PR^+^ HER2 1+ tumors. At the same time, the amount of miR-21 was high in ER- and PR-negative HER2-amplified and ER- and PR-negative HER2 1+ tumors. The miR-21 level in ER- and PR-negative HER2-amplified tumors was significantly higher than in ER^+^ and/or PR^+^ HER2-amplified BC.

In addition to miR-21, miR-423 and miR-200b were associated with the HER2 status. The levels of these miRNAs were higher in HER2-amplified tumors. The association with HER2 was also observed for miR-324, but only in tumors with Ki-67 < 14%. We found no association with ER and PR status for miR-200b and miR-193b; however, the levels of these miRNAs in ER^+^ and/or PR^+^ tumors with ER IHC scores of 0–5 differed significantly from miR-200b and miR-193b levels in tumors with higher ER expression. MiR-200b level was lower in tumors with ER expressions estimated at 0–5 IHC scores. According to previous studies, AR expression correlates with ER status [40]. We have also previously shown that in luminal B HER2 0 BC, AR level is higher for tumors with ER IHC scores of 6–8 compared to tumors with ER IHC scores of 0–5 [11]. Considering that miR-200b is an AR-regulated miRNA, the observed decrease in its levels in tumors with lower ER levels may be due to lower AR expression.

For miR-190a, we observed a decrease in expression in MCF-7 cells treated with progesterone. In ER^+^ and/or PR^+^ BC with high Ki-67 (≥ 14%), the miRNA level was significantly lower in tumor tissues with high PR expression (scores of 6–8) than in tissues of patients with lower receptor expression. Thus, a significant decrease in miR-190a expression in ER^+^ and/or PR^+^ BC can be caused by increased PR expression.

To test whether the identified miRNAs could be diagnostic markers in BC, we also analyzed the relation between their expression levels and tumor size, the presence of metastatic lymph node lesions, and the Ki-67 index. The levels of miR-193b, miR-324, miR-423, and miR-200b were associated with tumor size in ER^+^ and/or PR^+^ BC. MiR-193b level was higher in tissues of patients with tumors > 2 cm in HER2 0 BC; miR-324—in luminal B HER2-non-amplified; miR-423—in luminal A. MiR-200b expression was reduced in tumors > 2 cm compared to smaller tumors in BC with Ki-67 < 14%. Thus, the association of the miRNA level with tumor size depended on Ki-67 and HER2 expression.

The level of miR-190a was significantly reduced in the tissues of patients with LNM compared with cases without metastases in ER^+^ and/or PR^+^ BC with Ki-67 < 14%. That is, in tumors with Ki-67 > 14%, the miR-190a level depended on the PR level, while in tumors with Ki-67 < 14%, the level of miR-190a was associated with the LNM. The level of miR-193b was also lower in the tissues of patients with LNM in all ER^+^ and/or PR^+^ BC, except for BC with HER2 1+ status. Lower levels of miR-423 in tumor tissues of patients with LNM was observed in all luminal BCs excluding luminal B HER2 0. The most significant association with metastasis was for miR-193b and miR-190a. It was previously demonstrated that a decrease in miR-193b level can lead to an increase in cellular invasion in MDA-MB-231 and MDA-MB-435 cells, and miR-190 suppresses BC metastasis both in vitro and in vivo [41,42]. Thus, our results are consistent with previously obtained data. However, since we found that the miR-193b level in HER2-non-expressing tumors is associated not only with metastases, but also with tumor size, we assume that this miRNA is a more useful marker for predicting metastasis in luminal HER2-amplified cancer.

We have also previously shown miR-21 levels in HER2-expressing BC may be associated with the presence of LNM [11]. Here, when using a larger sample, we observed only a tendency to an increase in the miR-21 level in the tissues of patients with LNM compared to cases without, in HER2-expressing BC with Ki-67 ≥ 14%. In HER2-expressing BC with Ki-67 < 14%, we observed a tendency towards a decrease in the level of miR-21 in the tissues of patients with LNM. Further research is required on the relation between miR-21 and LNM in BC.

No association between miRNA levels and the presence of LNM was found for ER- and PR-negative tumors.

For miR-423, miR-200b, and miR-190b, we also found an association with Ki-67 in ER^+^ and/or PR^+^ BC. The levels of miR-423, miR-200b were higher in tissues with high Ki-67. The miR-190b level was also higher in BC tissues with Ki-67 values above the median in HER2-expressing tumors. However, in ER- and PR-negative HER2-amplified BC, miRNA expression, on the contrary, was lower in patients with Ki-67 above the median value. It is possible that this miRNA plays different roles in ER- and/or PR-positive BC and ER- and PR-negative BC. In addition, in TNBC, the miR-190a level was significantly lower in tissues with high Ki-67 (≥ 75%).

The levels of miR-27a and miR-21 were lower in the tissues of patients over the age of 50 compared to younger patients. Lower levels in tissues of patients over 50 years old were also observed for miR-190a—in luminal B HER2-amplified BC—and for miR-324—in ER^+^ and/or PR^+^ BC with Ki-67 < 14%. Menopause typically occurs between the ages of 49 and 52 years [43]. Therefore, the observed differences in the levels of miRNA in patients may be associated with hormonal changes after 50 years old.

Thus, we found that the expression of miR-190a, -190b, -27a, -193b, -324, -423, -200b, and -21, whose promoter regions contain binding sites for ER or AR, or putative binding sites for AR and PR, changes in MCF-7 cells when cells are treated with estradiol, progesterone, or testosterone. We have shown that the levels of these miRNAs may be associated with tumor size or the presence of lymph node metastases in breast cancer patients, but the presence of this association depends on the status and expression level of ER, PR, HER2, and Ki-67. As a result of our study we can draw the following conclusions: high expression levels of miRNA-190b in breast tumor tissue indicate a positive ER status; the assessment of miR-21, miR-423, and miR-200b levels could be used to confirm the amplification of HER2; and the levels of miR-190a (in ER+ and/or PR+ BC with Ki-67 < 14%), miR-193b (in luminal B HER2-amplified), and miR-423 (in luminal A, luminal B HER2 1+, and luminal B HER2-amplified tumors) can potentially be used as markers to predict the absence or presence of metastases in the lymph nodes.

## Figures and Tables

**Table 1:** Characteristics of the breast tumors under study.

Characteristics		ER- and/or PR-Positive (*n* = 156)	ER- and PR-Negative (*n* = 40)
Age (mean and range, year)		61 (27–90)	55 (38–76)
T stage	T1	71	18
	T2	81	20
	T3	2	1
	T4	2	1
N stage	N0	103	27
	N1	37	7
	N2	11	6
	N3	5	-
ER score	0–2	3	40
	3–5	7	-
	6–8	146	-
PR score	0–2	25	40
	3–5	34	-
	6–8	97	-
HER2 score	0	72	18
	1	47	5
	2–3	37	17

Estrogen and progesterone receptors (ER and PR). ER and PR were graded by the Allred scoring method [10].

**Table 2:** The miRNAs potentially regulated by ER, PR, and AR.

miRNA	ESR1 and ESR2 Binding Sites in Promoter According to ChipSeq Data (Homo Sapiens) [13]	ESR1 and ESR2 Binding Sites in Promoter According to Position Weight Matrix (Homo Sapiens) [13]	ESR1 and ESR2 Binding Sites in Promoter According to Position Weight Matrix (Mus Musculus and Rattus Norvegicus) [13]	AR/PR Binding Sites in Promoter According to Position Weight Matrix (Homo Sapiens)	AR/PR Binding Sites in Promoter According to Position Weight Matrix (Rattus Norvegicus)	Comments
hsa-mir-21	+	+	only mouse	+	+	It was demonstrated that androgen induced AR binding to the miR-21 promoter; MiR-21 expression was induced by R1881 in LNCaP and LAPC-4 cells [19]. Mibolerone inhibited basal expression of miR-21 in MCF-7 breast cancer cells [20]. Estradiol inhibited miR-21 expression in MCF-7 cells [21].
hsa-mir-190b	+	+	+	+	-	MiR-190b is the highest up-regulated miRNA in ER^+^ breast cancers compared to ER^−^ tumors. Did not observe an increase of miR-190b expression levels in MCF-7 or in T-47D treated by estradiol (1 nM for MCF-7 and 10 nM for T-47D, 6 h, 18 h, and 4 days) [22].
hsa-mir-200a/ hsa-mir-200b/ hsa-mir-429	+	+	+	-	-	MiR-200b showed the highest fold change under the influence of R1881 among androgen-sensitive miRNAs (PC3-AR cells) [23]. MiR-200b expression in MCF-7 cells decreased after 6 h of incubation with 10 nM estradiol [24].
hsa-mir-23a/ hsa-mir-24-2/ hsa-mir-27a	+	-	+	-	-	AR is able to associate transiently with the miR-23a/27a/24-2 promoter in response to androgen to initiate cluster transcription. The highest-fold change was observed for miR-27a and miR-23a (LNCaP cells treated with mibolerone) [25]. Estrogen induced miR-23a expression in SNU-387 cells [26].
hsa-mir-342	+	-	+	+	-	MiR-342 expression is positively correlated with ERα mRNA expression in human BC [27].
hsa-mir-190a	-	-	-	+	+	The promoter region of miR-190a contains half of an estrogen response element. ERα binds directly to this promoter [28]. Androgen inhibits miR-190a expression through direct binding to the half-site of ARE in miR-190a promoter (LNCaP cells) [29].
hsa-mir-378a	-	+	+	+	+	
hsa-mir-324	-	+	+	+	+	
hsa-mir-423	-	+	only rat	+	+	
hsa-mir-149	-	+	only rat	+	+	
hsa-mir-365b	+	+	only rat	+	-	
hsa-mir-574	+	-	only mouse	+	-	
hsa-mir-30a	+	-	only mouse	+	-	AR does not target the miR-30a promoter; AR activating signal may indirectly downregulate miR-30a (MDA-MB-453 cells) [30].
hsa-mir-10a	+	+	+	-	-	
hsa-mir-483	+	+	+	-	-	
hsa-let-7a-3/ hsa-let-7b	+	-	+	-	-	
hsa-mir-196a-2	+	+	-	-	-	MiR-196a expression is regulated by the estrogen receptor [31].
hsa-mir-33b	+	-	-	-	-	
hsa-miR-193b	-	+	+	+	-	Targets ER [32].
hsa-miR-126	-	+	+	+	-	Targets PR (regulation confirmed using mouse mammary epithelial cells) [33].

A plus signifies the presence of binding site in promoter region of miRNA according to the analysis performed. The list did not include miRNAs with low expression in breast tissues.

**Table 3:** Relative miRNA levels in MCF-7 cells treated with estradiol, testosterone, or progesterone.

miRNA	Time, h	Relative Level of miRNA					
		Estradiol		Testosterone		Progesterone	
		10 nM	100 nM	10 nM	100 nM	10 nM	100 nM
miR-23a	6	1.12	1.08	0.96	0.86	0.84	0.84
	24	0.89	0.95	0.88	1.00	0.92	0.88
	48	0.89	0.85	1.02	0.94	1.11	0.92
miR-27a	6	1.12	0.88	0.92	0.76 *	0.89	0.93
	24	1.07	1.10	1.09	1.08	0.93	0.92
	48	0.92	0.95	1.13	1.09	1.01	1.16
miR-190b	6	0.97	0.75 **	0.96	0.92	0.93	1.10
	24	1.35 *	1.37 **	1.13	1.09	1.02	1.03
	48	1.12	1.09	1.05	1.15	1.14	1.29 *
miR-190a	6	1.05	1.01	0.91	0.88	1.01	0.90
	24	1.09	1.02	0.90	0.99	0.79	0.75 *
	48	1.08	1.17	1.24 *	1.38 *	0.90	0.96
miR-200b	6	1.01	0.79 **	1.00	0.88	1.02	0.98
	24	0.98	1.01	1.10	0.99	0.91	0.96
	48	1.01	1.11	1.24 *	1.32 **	0.95	1.07
miR-21	6	1.03	0.98	0.92	0.62 **	1.17	1.36 *
	24	0.97	0.98	0.89	0.91	0.92	0.90
	48	1.07	1.08	1.36 **	1.40 **	1.11	1.08
miR-126	6	1.01	1.03	0.90	1.05	0.87	0.95
	24	0.94	0.88	1.06	0.94	1.01	1.12
	48	1.13	1.11	1.18	0.99	0.93	1.07
miR-378	6	1.09	1.00	0.88	0.94	0.90	1.08
	24	1.05	1.05	1.11	0.97	0.95	1.04
	48	1.00	1.17	1.15	1.19	0.94	1.12
miR-423	6	0.98	0.90	0.89	0.93	0.91	1.09
	24	1.04	1.06	0.99	0.87	0.96	0.99
	48	0.97	0.99	1.50 **	1.42 **	0.90	0.97
miR-149	6	1.25	1.01	0.95	0.91	0.96	1.06
	24	0.98	0.94	0.99	0.95	0.90	0.94
	48	0.89	1.05	1.05	0.89	1.02	1.18
miR-193b	6	1.07	1.00	0.93	0.95	0.92	1.07
	24	1.02	1.10	1.05	1.08	1.00	0.89
	48	1.24	1.30 **	1.30 **	1.40 **	1.07	1.08
miR-324	6	1.35 *	1.21	0.91	0.95	1.61 **	2.20 **
	24	0.99	1.06	1.06	1.05	1.05	1.04
	48	1.08	1.07	1.15	1.33 **	0.94	1.09
miR-342	6	0.83	0.96	0.89	1.10	0.92	1.12
	24	0.96	1.02	1.15	1.10	0.89	0.94
	48	1.07	1.08	1.17	1.19	0.90	1.18

Control cells were treated with vehicle (DMSO). Each value represents the mean of four independent experiments; the results are normalized to the control. The statistical significance of differences in miRNA expression in MCF-7 cells treated with compounds was calculated using the Student’s t-test. * *p* < 0.05 as compared with the control; ** *p* < 0.01 as compared with the control.

**Table 4:** Associations between the amounts of miR-190a, miR-190b, miR-27a, miR-193b, miR-324, miR-423, miR-200b, or miR-21 in tissue samples from BC patients and ER, PR, HER2 status, Ki-67 index, age, or LN status.

Characteristics		*n*	Relative Level * of miRNA and *p*-Value							
			miR-190a	*p*-Value	miR-190b	*p*-Value	miR-27a	*p*-Value	miR-193b	*p*-Value
ER and PR status	ER^+^ and/or PR^+^	156	0.10	0.165	4.84	**<0.001**	0.33	0.443	0.58	0.168
	ER^−^ and PR^−^	40	0.33		1.13		0.44		0.36	
HER2 status	HER2^+^	52	0.10	0.728	3.86	0.985	0.34	0.388	0.72	0.193
	HER2^−^	144	0.12		3.76		0.39		0.47	
Ki-67 index (%)	<14	65	0.11	0.753	3.89	0.941	0.49	0.394	0.49	0.567
	≥14	131	0.11		3.64		0.35		0.53	
Age	≤50	48	0.21	0.107	2.79	0.119	0.48	**0.045**	0.56	0.861
	>50	148	0.11		4.26		0.33		0.52	
N stage	N0	130	0.17	0.103	3.47	0.592	0.37	0.834	0.65	**0.022**
	N1-N3	66	0.09		4.21		0.42		0.37	
			**miR-324**	***p*-Value**	**miR-423**	***p*-Value**	**miR-200b**	***p*-Value**	**miR-21**	***p*-Value**
ER and PR status	ER^+^ and/or PR^+^	156	0.48	0.129	0.65	0.800	1.77	0.676	1.78	**0.004**
	ER^−^ and PR^−^	40	0.68		0.74		1.53		3.48	
HER2 status	HER2^+^	52	0.60	0.180	0.80	**0.004**	2.99	**0.024**	1.62	0.342
	HER2^−^	144	0.47		0.55		1.46		1.95	
Ki-67 index (%)	<14	65	0.39	0.257	0.56	**0.038**	1.26	**0.030**	2.02	0.947
	≥14	131	0.60		0.71		2.04		1.88	
Age	≤50	48	0.60	0.103	0.69	0.958	1.68	0.598	3.52	**0.003**
	>50	148	0.48		0.65		1.50		1.68	
N stage	N0	130	0.55	0.210	0.68	0.068	1.51	0.491	1.84	0.445
	N1-N3	66	0.48		0.52		1.78		2.13	

* Median of differences in miRNA levels between BC tissue and normal adjacent tissue (control) samples; the results were normalized to the control. Significant differences are highlighted in bold.

**Table 5:** Association of miR-190a, miR-190b, miR-27a, miR-193b, miR-324, miR-423, miR-200b, and miR-21 expression levels with clinicopathologic characteristics of ER- and/or PR-positive BC.

Characteristics		*n*	Relative Level * of miRNA and *p*-Value							
			miR-190a	*p*-Value	miR-190b	*p*-Value	miR-27a	*p*-Value	miR-193b	*p*-Value
**ER^+^ and/or PR^+^**										
T stage	T1	71	0.11	0.783	4.80	0.995	0.30	0.248	0.43	0.070
	T2–T4	85	0.09		4.93		0.46		0.74	
N stage	N0	103	0.13	0.058	4.57	0.477	0.37	0.391	0.69	**0.013**
	N1–N3	53	0.08		5.41		0.52		0.38	
Ki-67 index (%)	<M **	81	0.17	0.071	4.59	0.231	0.47	0.261	0.56	0.316
	≥M **	75	0.09		5.42		0.32		0.66	
ER score	6–8	146	0.09	0.475	5.04	0.078	0.32	0.113	0.54	**0.013**
	0–5	10	0.19		2.12		0.52		1.74	
PR score	6–8	97	0.09	**0.004**	4.71	0.840	0.29	**0.048**	0.49	0.258
	0–5	59	0.21		6.41		0.49		0.81	
Age	≤50	30	0.12	0.226	5.41	0.893	0.48	0.227	0.90	0.360
	>50	126	0.10		4.80		0.31		0.55	
			**miR-324**	***p*-Value**	**miR-423**	***p*-Value**	**miR-200b**	***p*-Value**	**miR-21**	***p*-Value**
**ER^+^ and/or PR^+^**										
T stage	T1	71	0.43	0.216	0.59	0.290	2.09	0.109	1.68	0.247
	T2–T4	85	0.50		0.67		1.36		1.81	
N stage	N0	103	0.53	0.313	0.68	**0.039**	1.64	0.832	1.74	0.802
	N1–N3	53	0.46		0.49		1.77		1.95	
Ki-67 index (%)	<M **	81	0.44	0.694	0.57	0.055	1.53	0.253	1.84	0.494
	≥M **	75	0.49		0.68		2.04		1.69	
ER score	6–8	146	0.48	0.467	0.62	0.756	1.88	**0.006**	1.69	0.106
	0–5	10	0.89		0.88		0.28		2.09	
PR score	6–8	97	0.45	0.211	0.63	0.600	1.85	0.858	1.76	0.336
	0–5	59	0.61		0.65		1.50		1.83	
Age	≤50	30	0.57	0.232	0.71	0.741	1.72	0.729	2.28	0.085
	>50	126	0.46		0.62		1.73		1.65	

* Median of relative differences in miRNA amounts between breast tumors and paired samples of normal adjoining (control) tissue; the results were normalized to the control. ** M—median value. For ER- and/or PR-positive BC, median = 18. Significant differences are highlighted in bold.

**Table 6:** Association of miR-190a, miR-190b, miR-27a, miR-193b, miR-324, miR-423, miR-200b, and miR-21 expression with clinicopathologic characteristics of ER- and PR-negative BC.

Characteristics		*n*	Relative Level * of miRNA and *p*-Value							
			miR-190a	*p*-Value	miR-190b	*p*-Value	miR-27a	*p*-Value	miR-193b	*p*-Value
T stage	T1	18	0.33	0.372	0.90	0.629	0.40	0.240	0.57	0.176
	T2–T4	22	0.37		1.26		0.57		0.35	
N stage	N0	27	0.37	0.824	1.07	0.835	0.39	0.183	0.44	0.725
	N1–N3	13	0.24		1.14		0.52		0.35	
Ki-67 index (%)	≤M **	19	0.39	0.162	1.45	0.191	0.45	0.779	0.47	0.272
	>M **	21	0.08		1.02		0.44		0.35	
Age	≤50	18	0.34	0.228	0.64	0.275	0.55	0.091	0.37	0.617
	>50	22	0.26		1.24		0.39		0.43	
			**miR-324**	***p*-Value**	**miR-423**	***p*-Value**	**miR-200b**	***p*-Value**	**miR-21**	***p*-Value**
T stage	T1	18	0.65	0.987	0.80	0.729	1.11	**0.036**	3.87	0.703
	T2–T4	26	0.75		0.51		2.04		3.47	
N stage	N0	27	0.69	0.391	0.70	0.969	1.33	0.349	3.33	0.101
	N1–N3	13	0.62		0.80		2.04		6.68	
Ki-67 index (%)	≤M **	19	0.89	0.101	0.79	0.546	1.34	0.706	3.48	0.582
	>M **	21	0.56		0.49		2.07		3.47	
Age	≤50	18	0.65	0.565	0.55	0.617	1.78	0.266	6.15	**0.019**
	>50	22	0.68		0.80		1.32		1.95	

* Medians of relative differences in miRNA levels between breast tumors and paired samples of normal adjoining (control) tissue; the results were normalized to the control. ** M—median value. For ER- and PR-negative BC, median = 40. Significant differences are highlighted in bold.

**Table 7:** The observed changes in the expression of miRNAs potentially regulated by ER, PR, or AR in MCF-7 cells treated with estradiol, progesterone, or testosterone.

miRNA	Regulation of miRNA Expression According to Bioinformatic Analysis	Observed Changes		
		MCF-7		
		Estradiol	Testosterone	Progesterone
miR-27a	ER *, AR *	-	down (6 h)	-
miR-190b	ER *, AR, PR	down (6 h) up (24 h)	-	up (48 h)
miR-190a	ER *, AR, PR	-	up (48 h)	down (24 h)
miR-200b	ER *, AR **	down (6 h)	up (48 h)	-
miR-21	ER *, AR *, PR	-	down (6 h) up (48 h)	up (6 h)
miR-423	ER, AR, PR	-	up (48 h)	-
miR-193b	ER, PR, AR	up (48 h)	up (48 h)	-
miR-324	ER, PR, AR	up (6 h)	up (48 h)	up (6 h)

* The presence of a binding site was previously confirmed by chromatin immunoprecipitation or reporter assay. ** The binding site has not been identified, but the sensitivity of miRNA to androgen has been previously demonstrated.

**Table 8:** The identified relation between miRNAs levels and tumor characteristics.

miRNA	Associated Tumor Characteristics
miR-27a	Age of the patients and the level of PR expression.
miR-190b	ER receptor status. Ki-67 index in ER^+^ and/or PR^+^ HER2-expressing, and Ki-67 index in ER^−^ and PR^−^ HER2-amplified tumors.
miR-190a	Expression level of PR. LN status in ER^+^ and/or PR^+^ tumors with low Ki-67. Ki-67 index in triple-negative tumors.
miR-200b	HER2 status and Ki-67 index. Tumor size in ER^+^ and/or PR^+^ tumors with low Ki-67. Level of ER expression in Luminal B tumors not expressing HER2.
miR-21	ER receptor status and age of the patients. Expression level of HER2.
miR-423	HER2 status and Ki-67 index. LN status in luminal A, luminal B HER2-amplified, and luminal B HER2 1+ tumors.
miR-193b	LN status in ER^+^ and/or PR^+^ tumors (except HER2 1+ cases). Tumor size in ER^+^ and/or PR^+^ HER2-non-expressing tumors. Expression level of ER in Luminal B HER2 0 tumors.
miR-324	Tumor size in Luminal B HER2-non-amplified tumors. Age of the patients and HER2 status in ER^+^ and/or PR^+^ tumors with low Ki-67.

## Data Availability

The data presented in this study are available on request from the corresponding author.
